# Living Ages

**Published:** 1859-04

**Authors:** 


### LIVING AGES.

It has been the aim of our *Journal* to inculcate the idea that man should be in his fullest mental prime at sixty, and ought to live in good health an hundred years, and so would we, as a general rule, if we lived wisely, temperately, every day. We expect to be living an hundred years to come, not bodily, but in influences. This journal is influencing its steady readers from month to month, to live more or less according to its teachings, giving them increased vigor of body, and of mind, to be perpetuated in their offspring and they again to theirs. This is what we call "living for ages."

Within a week, one of the best specimens of a whole man in New York, said of our writings, "they ought to be read, they will be read when you are gone." This single expression in the busiest hum of high noon in New York threw over our most time sunny heart, one of the most sudden and sombre clouds in our remembrance; not indeed a cloud of sorrow or of disapointment, but of responsibility, It came upon us like the weight of an avalanche, starting the enquiry, have I written truthfully? invitingly? Have I, in anything, hoisted a false light, which some foundering brother long afterwards looking trustfully to, shall mislead and make a wreck of?

There came the resolve, we will write more carefully hereafter, especially as our transient readers are more than five fold what they ever were before. The next moment our thoughts ran away off among our brother editors, and then all the writers and clergymen. Do they feel as fully as they ought, that every line they write, every sentiment they utter, are pebbles thrown on the bosom of the great sea of human life, which shall make waves of influences, that for all time, shall aid in propelling some human brother to glad successes, or to bitter disapointments, to final happiness, or to ultimate despair. Let us resolve then, one and all, as we must "live for ages," for good or for ill, that we will live to elevate and bless humanity, by being truthful in every line we write in every sentiment we utter.

